# The Effect of Palm Oil-Fried Street Kokor on Liver and Kidney Biomarkers of Swiss Albino Mice

**DOI:** 10.1155/2020/8819749

**Published:** 2020-12-04

**Authors:** Hailemariam Amsalu, Tesaka Wondimnew, Tigist Mateos, Minale Fekadie, Gesese Bogale

**Affiliations:** ^1^Department of Biomedical Sciences, Mizan-Tepi University, Ethiopia; ^2^Department of Biomedical Sciences, Jimma University, Ethiopia

## Abstract

**Background:**

Foods fried with oils at streets contain many harmful substances for health. Locally fried foods are consumed commonly in our society, yet their health effect is not studied.

**Objective:**

To assess the effect of palm oil-fried street kokor on liver and kidney biomarkers of Swiss Albino mice.

**Methods:**

Thirty-two male and female Swiss Albino mice with the age of 10-12 weeks old were divided randomly into four groups of eight members with equal male and female subgroups. The control group (group I) received only a standard pellet, and the experimental groups (group II, group III, and group IV) received 10%, 20%, and 30% kokor of their daily food consumption, respectively. At the end of the 6^th^ week, they were sacrificed by thoracoabdominal incision after anesthetizing by diethyl ether. Blood was taken from each mouse by cardiac puncture and analyzed for liver and kidney function tests.

**Result:**

The serum levels of liver damage biomarkers (alanine transaminase (ALT) and aspartate transaminase (AST)) and kidney damage biomarkers (urea and creatinine) of experimental groups were increased significantly relative to the control groups (*P* < 0.05). Level of biochemical profiles increased as the dose of kokor increased.

**Conclusions:**

Palm oil-fried street kokor damaged the liver and kidney of the mice, and the damage was exacerbated as the dose of kokor increased.

## 1. Introduction

Street foods are ready-to-eat foods and beverages prepared and/or sold by vendors on streets [1]. Among the street foods, fried foods are frequently consumed in many countries in different sanitary conditions and qualities [2–4].

Frying foods repeatedly changes the physical and chemical appearances of the oils. Some of the chemical reactions that occurred during the frying of oils are hydrolysis, oxidation, and polymerization [5, 6].

Hydrolysis reactions that occurred during frying of foods with oil result in the increment of free fatty acids, reactive oxygen species (ROS), and transfatty acids. These foods can induce organ failure and histopathology changes on different organs like the heart, intestinal mucosa, liver, and kidney [7, 8]. Correspondingly, oxidation of oils during frying alters the nature of enzymes and the status of antioxidants and causes the formation of lipid peroxidation and transfatty acids [9, 10]. In addition, polymerization reactions form total polar compounds like triglyceride dimer (TGD), triglyceride oligomer (TGO), aldehyde by-products, alcohol, hydrocarbons, and acrolein [11, 12], and these substances cause lipid deposition, oxidative stress, and cytotoxicity by deactivating genes of enzymes which are important for lipolysis [13].

The cellular damage of fried foods is dependent on the dose of fried foods eaten. Consumption of large amounts of fried and snack foods disturbs lipid metabolism and induces oxidative stress and eventually creates a toxic environment for cells [10].

Vegetable oils are used for frying foods [14]. Palm oil, a known frying vegetable oil in Ethiopia, consumption is increasing rapidly in the past several decades because of its cheap cost and good frying properties [15]. Different studies at different settings proved that heating palm oil at different frequencies and temperature values causes deleterious effects on consumers' health [16].

Since repeatedly fried oils lose their ability to scavenge free radicals, palm oil loses its antioxidant ability as its duration and frequency of frying increase. Antioxidants such as *α*-tocopherol and *γ*-tocotrienol within palm oil are degraded faster than those in other oils due to more oxidation of fatty acids [17].

Chronic feeding of oxidized palm oil induces organ damages and dysfunctions. Repeatedly and long-time heated palm oil causes the damage of liver tissues [16]. As the liver is a major organ involved in energy metabolism, the effects of oxidized edible oils on the liver is tangible [18]. The heated palm oil causes the increment of liver function enzymes, extensive hepatocyte death, and chronic liver disease [18, 19]. It also causes decreases in renal plasma flow and glomerular filtration rate. It also causes the elevation of both systolic and diastolic blood pressures which cause glomerular injury [20].

Even though different fried foods are prepared in Ethiopia in different setups, we have not got a single study that examined the health effects of these foods. This study tried to investigate the effect of palm oil-fried street kokor on the liver and kidney biomarkers of Swiss Albino mice.

## 2. Hypotheses of the Study

- Palm oil-fried street kokor has no significant effect on liver and kidney biomarkers of Swiss Albino mice
- Sex has no significant effect on liver and kidney biomarkers of Swiss Albino mice
- Palm oil-fried street kokor and sex have no significant interaction on the liver and kidney biomarkers of Swiss Albino mice

## 3. Methods and Materials

### 3.1. Study Design and Sample Size Determination

A laboratory-based randomized control experimental trial was conducted on thirty-two Swiss Albino mice. The sample size was determined based on resources [21, 22].

### 3.2. Experimental Animals and Their Grouping

**Figure 1:**
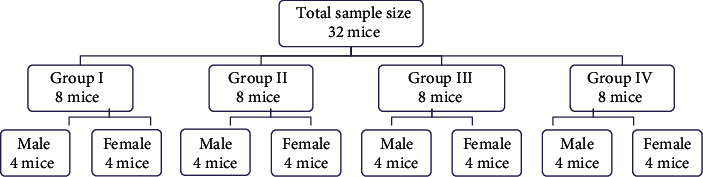
Random allocation of Swiss Albino mice into groups for the experiment.

Thirty-two (16 males and 16 females) Swiss Albino mice, with the age ranging from 10 to 12 weeks, were obtained from the Jimma University Tropical and Infectious Disease Research Center, Sokoru, Ethiopia. They were brought to the Jimma University Veterinary Medicine Postgraduate Laboratory and free to access food and distilled water ad libitum per the National Institutes of Health (NIH) Guidelines for Care and Use of Laboratory Animals [23]. The mice were allowed to share the same environmental condition within a ventilated room with humidity of 60%-70% and temperature of 20-26°C. They were acclimatized for one week before the beginning of the experiment and at a 12-hour light/dark cycle throughout the study. Then, the mice were divided randomly into 4 groups, having eight members each. Each group was also again categorized randomly into two subgroups as male and female subclasses in which each subclass (four mice of a similar sex) was in different stainless steel and plastic cages with dimensions of length 40 cm, width 20 cm, and height 15 cm, and the cages were cleaned daily (see Figure 1).

### 3.3. Experimental Diet Preparation and Dosing Procedures

Palm oil (3 l), wheat flour (5 kg), and commercial yeast were purchased from local markets in Jimma Town, Ethiopia. The kokor was prepared based on local procedures in collaboration with street food vendors at four different streets of Jimma Town. Group I (the control group) received only a standard pellet, and the experimental groups (groups II, III, and IV) received 10%, 20%, and 30% kokor of their daily food consumption, respectively, according to their body weight [19, 24]. This is due to the fact that fried foods constitute 7% and 21% of the total food consumed per day in Spain and India, respectively [10, 25]. Mice eat 10% of their body weight per day [26]. For the sake of administering the kokor easily, the daily total dose of each mouse was divided into two equal doses and dissolved in distilled water in a concentration of 300 mg/ml and each half dose was given in 12-hour intervals through a 24-gauge oral gavage needle for six weeks [10, 27]. The dose of the experimental diet was calculated by the following formula.

(1) $$\begin{array}{c} D=\frac{\text{BW}\times 10}{100}\times K, \end{array}$$

where *D* is the dose of kokor given to each mouse per day (mg), BW is the body weight of each mouse (g), 10/100 is the daily food consumption of mice relative to their body weight, and *K* is the proportion of kokor given to each mouse in a specific group (%).

### 3.4. Data Collection Procedures

The weight of the mice was measured every week to evaluate the effect of kokor on body weight and registered using a checklist. At the end of the 6^th^ week, they were fasted for 12 hours and sacrificed by thoracoabdominal incision after asphyxiating by diethyl ether [28]. Two to two and a half milliliter (2-2.5 ml) of blood was taken from each mouse through cardiac puncture and collected with a plain tube containing serum separator tube (SST) gel. The serum was separated through centrifugation with a speed of 3000 revolutions per minute at room temperature for 10 minutes and put in an ice box and finally analyzed for liver function tests (ALT and AST) and kidney function tests (urea and creatinine).

### 3.5. Data Quality Assurance

Before, during, and after the analysis, precautions were considered and the equipment was calibrated. All manipulations and procedures conducted on the samples were done by trained professionals. Liver function tests (AST, ALT) and kidney function tests (urea and creatinine) were analyzed by using the ABX Pentra 400 clinical chemistry autoanalyzer (HORIBA ABX SAS, China) as per the manufacturer's instructions (Annex I).

### 3.6. Data Analysis Procedures

Data were entered into EpiData software version 3.1 and then exported to SPSS version 25.0 software for analysis after it was checked and cleaned. Results were presented by tables and figures and expressed as mean ± SD. Statistical data analysis was done using two-way ANOVA post hoc multiple comparisons (Tukey test), and *P* ≤ 0.05 was considered statistically significant.

### 3.7. Ethical Considerations

The research was conducted after getting an ethical approval letter from the Jimma University Institutional Review Board with a reference no. of IHRPGD/680/2019, and a support letter was written for the College of Agriculture and Veterinary Medicine of the university.

## 4. Results and Discussion

Thirty-two Swiss Albino mice (16 males and 16 females) with the age ranging from 10 to 12 weeks were evaluated in the study. Their initial mean weight was 37.40 ± 2.00 g, and the final mean average weight was 38.30 ± 3.37 g.

### 4.1. Liver Function Tests

Serum ALT and AST levels are among the current indicators of the damage of the liver and hepatotoxicity [29]. When liver tissue is damaged, ALT and AST are released into the bloodstream and the level of the enzymes rises in serum. So, the amount of ALT and AST in the blood is directly associated with the number of tissue damage [30].

The two-way ANOVA results showed that there was no statistically significant interaction between the effect of the dose of the palm oil-fried street kokor and sex on serum ALT level (*F* [3, 24] = 0.069, *P* = 0.976) and serum AST level of the mice (*F* [3, 24] = 0.156, *P* = 0.925). In the same way, the sex of the mice had no significant effect on the serum ALT level (*F* [1, 24] = 1.291, *P* = 0.265) and serum AST level of the mice (*F* [1, 24] = 3.125, *P* = 0.089). On the other hand, the dose of the palm oil-fried street kokor had a significant effect on the serum ALT level (*F* [3, 24] = 122.022, *P* = 0.001] and serum AST level of the mice (*F* [3, 24] = 260.285, *P* = 0.001).

#### 4.1.1. ALT

Alanine aminotransferase (ALT, EC 2.6.1.2) is the most frequently relied upon laboratory indicator of hepatotoxic effects [29].

In this study, the serum ALT level of the experimental groups was increased significantly when compared with that of the control group (*P* < 0.05). Likewise, the serum ALT level increased significantly among experimental groups as the dose of the palm oil-fried kokor increased (*P* < 0.05) (see Table 1). The finding of this study supports the result of a study done by Imafidon and Okunrobo on rats which indicated that the serum level of ALT is increased for subjects who have taken oxidized palm oil in different forms and doses [31].

#### 4.1.2. AST

Aspartate aminotransferase (AST, EC 2.6.1.1) is another indicator of liver damage but less specific as it is found in other organs like the heart, brain, skeletal muscle, and liver tissue [32].

Alike to ALT, the serum AST level of the experimental groups was significantly increased when compared with that of the control groups (*P* < 0.05). Similarly, the serum AST level increased significantly among experimental groups as the dose of the palm oil-fried kokor increased (*P* < 0.05) (see Table 1). The finding of this study supports the result of other previously done studies by Li et al. and Imafidon and Okunrobo on rats and rabbits, respectively, which indicated that the serum level of AST is increased for subjects who have taken oxidized palm oil in different forms and doses [18, 31].

The elevation of the two enzymes in the serum is an indicator of liver tissue damage [32]. The damage of the liver might be related to the formation of toxic substances formed during the frying process of the street kokor by palm oil [10].

### 4.2. Kidney Function Tests

Urea and creatinine are the most frequently ordered tests to examine kidney function. Urea is produced by the breakdown of proteins and is excreted in urine whereas creatinine is a nonprotein nitrogenous compound that is produced by the breakdown of creatine in muscle and excreted by glomerular filtration at a constant rate [33, 34].

In this study, there was no statistically significant interaction between the effect of the dose of the palm oil-fried street kokor and sex on serum urea level (*F* [3, 24] = 0.068, *P* = 0.977) and serum creatinine level of the mice (*F* [3, 24] = 0.049, *P* = 0.985). In the same way, the sex of the mice had no significant effect on the serum urea level (*F* [1, 24] = 0.575, *P* = 0.455) and serum creatine level of the mice (*F* [1, 24] = 0.763, *P* = 0.391). On the other hand, the dose of the palm oil-fried street kokor had a significant effect on the serum urea level (*F* [3, 24] = 21.231, *P* = 0.001) and serum creatine level of the mice (*F* [3, 24] = 372.775, *P* = 0.001).

#### 4.2.1. Serum Urea

As described in Table 2, the serum urea and creatinine level of the experimental group mice were significantly increased when compared with those of the control groups (*P* < 0.05). Similarly, the serum urea level of group IV was increased significantly when compared with the serum urea level of group II mice (*P* < 0.05).

#### 4.2.2. Serum Creatinine

There was a significant increment of serum creatinine levels of experimental group mice when compared with the control group mice (*P* < 0.05). In addition, the serum creatinine level of group IV mice was higher than the serum creatinine level of group II and group III mice significantly (*P* < 0.05). Similarly, the serum creatinine level of group III mice was significantly higher than the serum creatinine level of group II mice (*P* < 0.05).

The findings of this study are similar to the results of the previously done studies on rats which emphasize the harmful effect of oxidized palm oil on the kidney [7, 19, 35]. The damage of the kidney of the mice could be due to lipid peroxidation and the free radicals formed during the frying process of the kokor with palm oil [20, 36].

## 5. Conclusions

Palm oil-fried street kokor damaged the liver and kidney of the mice. The harmful effect of the kokor was exacerbated as the dose of the fried kokor increased. The damaging effect of the food was approved through biochemical evaluations. The liver enzymes (ALT and AST) and the kidney function tests (urea and creatinine) increased significantly in the serum of the palm oil-fried street kokor-fed mice which are the manifestations of liver damage.

## Figures and Tables

**Table 1:** Serum level of ALT and AST enzymes of the groups of the male and female mice.

Groups	ALT (U/l)		AST (U/l)	
	Male	Female	Male	Female
Group I	23.60 ± 2.47^a^	23.72 ± 2.18^a^	78.54 ± 1.80^a^	81.59 ± 3.10^a^
Group II	35.08 ± 2.70^b^	35.63 ± 4.33^b^	99.44 ± 6.59^b^	103.90 ± 3.47^b^
Group III	40.92 ± 2.38^c^	43.56 ± 4.35^c^	121.48 ± 5.45^c^	122.72 ± 7.56^c^
Group IV	51.82 ± 3.49^d^	52.25 ± 1.38^d^	145.75 ± 6.51^d^	149.71 ± 2.75^d^

The results were expressed as mean ± SD. Values with different superscripts within the same column are statistically significant (*P* < 0.05).

**Table 2:** Serum urea and creatinine level of the groups of the male and female mice.

Groups	Urea (mg/dl)		Creatinine (mg/dl)	
	Male	Female	Male	Female
Group I	39.63 ± 0.33^a^	39.59 ± 0.22^a^	0.56 ± 0.02^a^	0.58 ± 0.01^a^
Group II	40.24 ± 0.44^b^	40.19 ± 0.45^b^	0.75 ± 0.02^b^	0.74 ± 0.02^b^
Group III	40.52 ± 0.34^bc^	40.45 ± 0.13^bc^	0.82 ± 0.03^c^	0.81 ± 0.01^c^
Group IV	40.86 ± 0.21^c^	40.69 ± 0.09^c^	0.84 ± 0.02^c^	0.83 ± 0.02^c^

The results were expressed as mean ± SD. Values with different superscripts within the same column are statistically significant (*P* < 0.05).

## Data Availability

The data used to support the findings of this study are included within the supplementary information files.

## Supplementary Materials

Supplementary Materials File I: laboratory results of the serum of the mice.
