# Supplementary material for: The Effect of Palm Oil-Fried Street Kokor on Liver and Kidney Biomarkers of Swiss Albino Mice
Source: J Lipids. 2020 Dec 4;2020:8819749. doi: 10.1155/2020/8819749 (PMC7787862; doi:10.1155/2020/8819749)
Supplement: Supplementary Materials — File I: laboratory results of the serum of the mice. [file 8819749.f1.zip › File 1.pdf]

File I

Laboratory results of the serum of the mice

Ser No.	Group	Sex	ALT (U/L)	AST (U/L)	Urea (mg/dl)	Creatinine (mg/dl)
1	1	1	27.12	80.61	39.24	0.59
2	1	1	21.61	79.21	40.02	0.61
3	1	1	22.22	76.40	39.54	0.59
4	1	1	23.45	77.92	39.72	0.56
9	2	1	31.60	93.47	39.78	0.73
10	2	1	34.52	102.29	40.02	0.74
11	2	1	37.91	107.39	40.38	0.76
12	2	1	36.29	94.62	40.80	0.77
17	3	1	38.41	115.37	40.44	0.81
18	3	1	42.26	128.46	40.08	0.79
19	3	1	43.53	119.91	40.68	0.80
20	3	1	39.47	122.18	40.88	0.78
25	4	1	49.84	142.11	40.74	0.83
26	4	1	47.90	138.42	40.62	0.83
27	4	1	53.76	151.23	41.04	0.85
28	4	1	52.76	151.23	41.04	0.85
5	1	2	21.70	83.68	39.30	0.58
6	1	2	23.85	82.82	39.54	0.59
7	1	2	26.71	76.97	39.78	0.58
8	1	2	26.61	82.89	39.72	0.57
13	2	2	39.91	99.57	39.60	0.72
14	2	2	31.27	105.56	40.32	0.74
15	2	2	32.61	102.86	40.14	0.73
16	2	2	38.74	107.58	40.70	0.78
21	3	2	38.39	126.17	40.36	0.79
22	3	2	41.90	111.96	40.44	0.80
23	3	2	42.57	129.31	40.65	0.80
24	3	2	48.39	123.45	40.38	0.77
29	4	2	50.60	148.29	40.58	0.81
30	4	2	53.74	153.69	40.72	0.84
31	4	2	51.70	149.31	40.68	0.85
32	4	2	52.96	147.56	40.80	0.85

Key for sex
1 = Male
2 = Female
