# Supplementary material for: The Effect of Palm Oil-Fried Street Kokor on Liver and Kidney Biomarkers of Swiss Albino Mice
Source: J Lipids. 2020 Dec 4;2020:8819749. doi: 10.1155/2020/8819749 (PMC7787862; doi:10.1155/2020/8819749)
Supplement: Supplementary Materials — File I: laboratory results of the serum of the mice. [file 8819749.f1.zip › SUPPLEMENTARY FILE DESCRIPTION.docx]

SUPPLEMENTARY FILE DESCRIPTION:

**File I : Laboratory results of the serum of the mice**
